# Women’s psychosocial outcomes following an emergency caesarean section: A systematic literature review

**DOI:** 10.1186/s12884-019-2687-7

**Published:** 2019-12-30

**Authors:** Madeleine Benton, Amy Salter, Nicole Tape, Chris Wilkinson, Deborah Turnbull

**Affiliations:** 1 School of Psychology, University of Adelaide, Adelaide, South Australia Australia; 2 School of Public Health, University of Adelaide, Adelaide, South Australia Australia; 3 Maternal Fetal Medicine, Women’s and Children’s Hospital, Adelaide, South Australia Australia

**Keywords:** Systematic literature review, Childbirth, Emergency caesarean section, Psychosocial outcomes, Maternal health, Postpartum

## Abstract

**Background:**

Given the sudden and unexpected nature of an emergency caesarean section (EmCS) coupled with an increased risk of psychological distress, it is particularly important to understand the psychosocial outcomes for women. The aim of this systematic literature review was to identify, collate and examine the evidence surrounding women’s psychosocial outcomes of EmCS worldwide.

**Methods:**

The electronic databases of EMBASE, PubMed, Scopus, and PsycINFO were searched between November 2017 and March 2018. To ensure articles were reflective of original and recently published research, the search criteria included peer-reviewed research articles published within the last 20 years (1998 to 2018). All study designs were included if they incorporated an examination of women’s psychosocial outcomes after EmCS. Due to inherent heterogeneity of study data, extraction and synthesis of both qualitative and quantitative data pertaining to key psychosocial outcomes were organised into coherent themes and analysis was attempted.

**Results:**

In total 17,189 articles were identified. Of these, 208 full text articles were assessed for eligibility. One hundred forty-nine articles were further excluded, resulting in the inclusion of 66 articles in the current systematic literature review. While meta-analyses were not possible due to the nature of the heterogeneity, key psychosocial outcomes identified that were negatively impacted by EmCS included post-traumatic stress, health-related quality of life, experiences, infant-feeding, satisfaction, and self-esteem. Post-traumatic stress was one of the most commonly examined psychosocial outcomes, with a strong consensus that EmCS contributes to both symptoms and diagnosis.

**Conclusions:**

EmCS was found to negatively impact several psychosocial outcomes for women in particular post-traumatic stress. While investment in technologies and clinical practice to minimise the number of EmCSs is crucial, further investigations are needed to develop effective strategies to prepare and support women who experience this type of birth.

## Introduction

There has been a dramatic increase in caesarean section (CS) rates around the world over the past three decades, particularly in middle and high income countries [1]. At a population level, the World Health Organization has concluded that CS rates higher than 10% are not associated with reductions in maternal and newborn mortality rates [2]. Despite this, recent data has reported rates of 40.5% in Latin America and the Caribbean, 32.3% in Northern America, 31.1% in Oceania, 25% in Europe, 19.2% in Asia and 7.3% in Africa [3]. Globally, CS rates have almost doubled between 2000 and 2015, from 12 to 21% [4].

CSs are broadly classified depending on whether they are an elective or emergency procedure. An elective CS is defined as a planned, non-emergency delivery which occurs before initiation of labour [5]. In contrast, emergency caesarean section (EmCS) is defined as an unplanned CS delivery performed before or after onset of labour, which is typically urgent and is most often required due to fetal, maternal or placental conditions (eg. fetal distress, eclampsia, placental/cord accidents, uterine rupture, failed instrumental birth etc) [5, 6].

While CS has an important place in potentially protecting both mother and baby from harm, it is associated with short and long term physical and psychological risks which can extend many years beyond the current delivery and effect the health of the woman, her child, and future pregnancies [7]. In a review of research on the outcomes of CS, Lobel [8] noted that the procedure is uniquely challenging as it combines surgery and birth, events that elicit very diverse emotional responses. The circumstances surrounding an EmCS add an additional layer of complexity to this experience which has thereby prompted researchers to explore the psychosocial impact of this type of birth. The nature of the event accompanied by a series of subsequent rapid psychological adjustments may be distressing, anxiety-provoking and emotionally unsettling for women [9, 10].

The primary outcome of obstetric care, is of course, to ensure both mother and infant remain physically healthy however, psychosocial aspects and outcomes of maternity care and obstetrics are no less important [11, 12]. Psychosocial outcomes identified and examined in the literature as potentially related to CS include: mental health problems such as, postpartum depression, post-traumatic stress and anxiety; decreased maternal satisfaction with childbirth; the mother infant relationship; parents’ sexual functioning; and health behaviours such as infant feeding.

### The current study

Given the nature of EmCS and the increased risk of psychological distress for women, it is imperative to gain insight into the diverse psychosocial outcomes for women experiencing this type of birth. Knowledge and awareness surrounding the impact of EmCS on women’s psychosocial outcomes is likely to enhance the overall quality of maternity care. The aim of the current systematic literature review is to identify, collate, and examine the evidence surrounding women’s psychosocial outcomes of EmCS.

## Method

A systematic literature review constituting a rigorous method of research for summarising evidence from multiple studies on a specific topic was undertaken [13, 14]. The present study was conducted in accordance with the Preferred Reporting Items for Systematic Reviews and Meta-analyses (PRISMA) recommendations [15]. An a priori designed study protocol guided the literature search, study selection and data synthesis, with quantitative meta-analysis attempted when possible. This systematic review was registered in the international prospective register of systematic reviews (PROSPERO) database: CRD42018087677.

### Search strategy

The search strategy was designed and developed following consultation with a health and medical sciences university librarian in order to ensure a comprehensive search and increase the robustness of the study [16]. The medical and psychological electronic databases of EMBASE, PubMed, Scopus, and PsycINFO were searched between November 2017 and March 2018. When conducting searches, keywords were combined representing the two primary concepts; psychosocial outcomes and EmCS. In this systematic literature review, psychosocial outcomes were considered to be variables that encompass social and psychological aspects of an individual’s life [17]. The Boolean operators ‘OR’ and ‘AND’ were utilised to facilitate maximum inclusion of relevant articles [18]. Detailed search algorithms and indexing language used for each database are outlined in the Additional File 1.

To ensure that included articles were reflective of original and recently published research, limits were applied within the literature search to incorporate inclusion criteria such as: research articles, publication within the last 20 years (1998 to 2018), and peer-reviewed articles [19]. Further, the search was limited to English language publications due to unavailability of funding for language translation. Grey literature or trial registries were not persued for practical purposes.

### Eligibility criteria

Inclusion and exclusion criteria (based on the PICOS [population, intervention, comparison, outcome, study design] framework) were established in advance and documented in the review protocol to identify all pertinent studies.

- **P**opulation: Women who have delivered via EmCS
- **I**ntervention: EmCS
- **C**omparison: Any mode of delivery (MoD) where reported, otherwise no comparison
- **O**utcomes: Psychosocial variables (i.e. postnatal depression, anxiety, post-traumatic stress, infant feeding, sexual functioning, satisfaction, views and experiences)
- **S**tudy Design: Quantitative (excluding case studies), qualitative or mixed methods

### Study selection

Potential papers were screened initially by title and abstract by two reviewers who reviewed half of papers each (MB and NT) and full texts were retrieved for those citations considered potentially relevant for inclusion. Both reviewers completed an initial subset of papers together in order to ensure consistency in their approach. Reference lists of retrieved full text papers were examined to identify potentially relevant studies not captured by electronic searches [20]. Full texts of the remaining articles were independently appraised against the eligibility criteria for final inclusion by two reviewers (MB and NT). In case of disagreement in the selection process, a third reviewer was available for consultation.

### Data extraction

Utilising a data extraction form designed by the authors, MB extracted descriptive data on study aims, study design, study location, sample size, data collection period, measures utilised, and included a text description summarising the psychosocial and EmCS related findings from each study. These data were cross-checked by NT. A data synthesis of the findings from each article was then performed, involving identification of prominent and recurrent themes in the literature and the synthesis of findings from studies under thematic headings. This approach has been described as flexible, allowing considerable latitude to systematic reviewers, and provides a means of integrating qualitative and quantitative evidence [20].

### Quality assessment

In line with standard systematic literature review methodology a formal methodological quality appraisal of each included study was performed using the Mixed Methods Appraisal Tool (MMAT) version 11 [21]. This tool allows for the critical appraisal of quantitative, qualitative, and mixed methods studies and was developed to address some of the challenges of critical appraisal in systematic mixed studies reviews. The MMAT has been validated and used for quality assessment in similar mixed method systematic reviews [22]. The MMAT comprises 19 items for appraising the methodological quality of 5 different types of studies: qualitative studies (4 items), randomised controlled trials (4 items), non-randomized studies (4 items), quantitative descriptive studies (4 items), and mixed methods studies (4 items). Based on the number of criteria met for an individual study, the overall quality assessment rating (QAR) is presented using descriptors *, **, ***, and ****, ranging from * (single criterion met) to **** (all criteria met). Each study included in the quality assessment was evaluated by two independent reviewers (MB and NT). A third reviewer was available for consultation if disagreement occurred.

## Results

### Study selection and characteristics

**Fig. 1:**
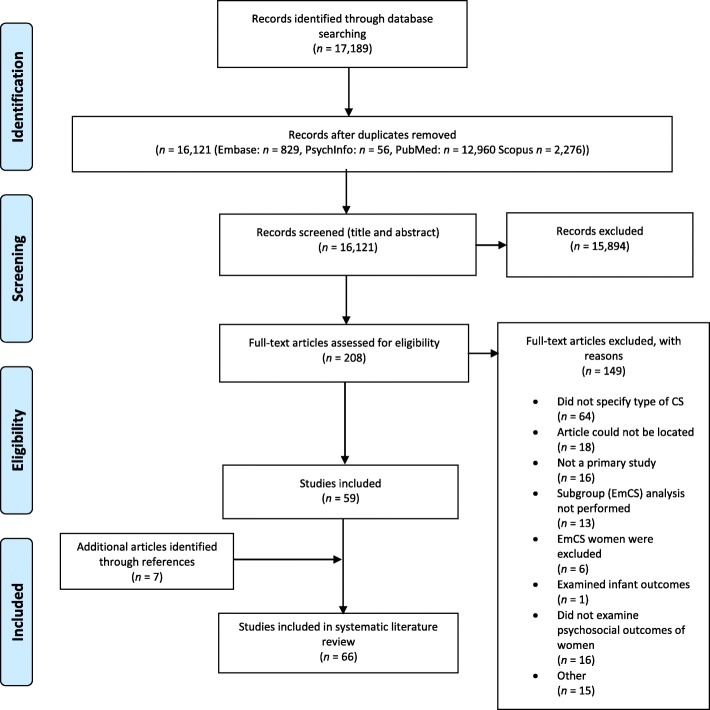
Search and Selection Flow Diagram

A summary of the search process is illustrated in Fig. 1, as recommended by the PRISMA guidelines [15]. In total 17,189 articles were initially identified. For the initial screening, all search results were imported into citation management software Endnote × 7 where 1068 duplicates were identified and removed, leaving 16,121 articles (Pubmed, *n* = 12,960, EMBASE *n* = 829, PsycINFO *n* = 56, Scopus *n* = 2276). Titles and abstracts were then assessed by two reviewers (MB, NT), with this process ending with the inclusion of 208 articles. Full texts were then retrieved for those citations considered potentially relevant and assessed for eligibility by the two reviewers (MB, NT). Of these 208 articles, 149 were excluded. The most common reason for exclusion was a lack of differentiation between type of CS when reporting study results (see Fig. 1). Reference lists of included studies were hand searched by the first author and a further 7 articles were subsequently included. A total of 66 relevant articles [5, 9, 23–86] were thus included in the current systematic literature review.

### Description of included studies

Characteristics of the 66 included studies are presented in Table 1. Studies were conducted in 22 different countries with the majority conducted in Sweden (*n* = 12), followed by the UK (*n* = 10), and then Nigeria (*n* = 5). Most studies were quantitative in nature (*n* = 51), followed by qualitative (*n* = 14) and just one study with mixed methods. Cross sectional (*n* = 19) and prospective designs (*n* = 31) were most prevalent.

**Table 1:** Summary characteristics of included studies

Author/Year	Aim	Study Design	Study Location	Participants	Time frame	Study Period	Measure	Psychosocial Outcomes	Relevant Key Findings	MMAT QAR^a^
Adams, 2012	To assess the association between mode of delivery (MoD) and maternal postpartum emotional distress.	Prospective Cohort	Norway	55, 814	17 & 30 weeks gestation and 6 months postpartum	1998–2008	Short form of the Hopkins Symptom Checklist-25 (SCL-8)	Emotional Distress	MoD was not associated with the presence of emotional distress postpartum.	*****
Adewuya, 2006	To estimate the prevalence PTSD after childbirth and to examine associated factors.	Cross-sectional	Nigeria	876	6 weeks postpartum	2004	MINI International Neuropsychiatric Interview, Index of marital satisfaction, Medical Outcomes Study Social Support Survey, Life events scale, Labour agentry scale	PTSD	Instrumental delivery and Emergency Caesarean Section (EmCS) were associated with PTSD, while elective caesarean section (ElCS) sections showed no significant effect.	*****
Ahluwalia, 2012	To assess the relationship between MoD and breastfeeding.	Prospective longitudinal	United States	3026	Before birth and 10 times during the year after birth.	2005–2006	Study specific	Breastfeeding	Median breastfeeding duration was 20.6 weeks for EmCS. Breastfeeding duration among women who initiated breastfeeding show that the prevalence of breastfeeding at any time through 60 weeks after delivery was lowest for those who had induced VD or EmCS than among those in the other two groups (spontaneous VD or planned CS).	
Beck, 2008	To explore the impact of birth trauma on mothers’ breast feeding experiences.	Qualitative	New Zealand, US, Australia, UK, Canada	52	Unspecified	Unspecified	Study specific	Infant feeding	Women repeatedly explained that their decision to breastfeed was driven by their need to make amends to the infants for the traumatic way they had arrived into the world, for example, by EmCS.	*****
Baas, 2017	To understand the relationship between client-related factors and the experience of midwifery care during childbirth to improve care.	Prospective longitudinal	Netherlands	2377	20 and 34 weeks pregnant and 6 weeks postpartum	2009–2011	Study specific and Labour Agency Scale	Experience of care	MoD effected experiences of care. Women who had an unplanned CS were more likely to indicate that they had received “less than good” midwifery care during childbirth.	****
Baston, 2008	To examine what factors relate to women’s appraisal of their birth three years later.	Prospective Cohort	England and Netherlands	2048	3 years postpartum	2003–2004	Study specific	Satisfaction of experience	EmCS was a factor contributing to a negative appraisal of birth in England and the Netherlands.	****
Bergant, 1998	To study the subjective psychological and physical stressful experience of childbirth burden.	Cross-sectional	Austria	1250	5 days postpartum	1993–1994	EPDS, Trait-Anxiety Inventory, Burden of childbirth	Burden of childbirth	Women who experienced emergency surgical intervention (EmCS and vacuum extraction) demonstrated higher childbirth burden scores.	****
Bryanton, 2008	To determine factors that predict women’s perceptions of the childbirth experience and to examine whether these vary with the type of birth a woman experiences.	Prospective cohort	Canada	652	12–47 h postpartum	2004–2005	Questionnaire Measuring Attitudes About Labour and Delivery	Perceptions of birth	Women who had a planned CS birth scored significantly lower on birth perception than those who had an EmCS or a VD.	****
Burcher, 2016	To elicit women’s narratives of their unplanned CS births to identify potentially alterable factors that contribute to CS regret.	Qualitative	United States	14	2–6 weeks postpartum	Unspecified	Study specific	Regret and dissatisfaction	Four key themes emerged from patients’ unplanned CS narratives: poor communication, fear of the operating room, distrust of the medical team, and loss of control.	*****
Carquillat, 2016	To compare subjective childbirth experience according to different delivery methods.	Cross-sectional	Switzerland and France	291	4–6 weeks postpartum	2014–2015	Questionnaire for Assessing Childbirth Experience	Childbirth Experience	Women who had an EmCS were at highest risk of experiencing childbirth in a negative way.	****
Chen, 2002	To compare women who had a VD with those who had a CS in depression, perceived stress, social support, and self-esteem.	Cross-sectional	Taiwan	357	6-weeks postpartum	1999	The Beck Depression Inventory, The Perceived Stress Scale, The Interpersonal SupportEvaluation List (ISEL) Short Form, Coopersmith’s Self-Esteem Inventory	Depression, perceived stress, social support, self-esteem	There was no association found in this study between the type of CS (planned or emergency) and psychosocial measures.	*****
Creedy, 2000	To determine the incidence of acute trauma symptoms and PTSD in women as a result of their labour and birth experiences, and to identify factors that contributed to the women’s psychological distress.	Prospective Longitudinal	Australia	499	4–6 weeks postpartum	1997–1998	Posttraumatic Stress Symptoms interview	PTSD	The experience of an EmCS was correlated with the development of trauma symptoms.	****
Durick, 2000	To examine if unplanned CS would be related to less optimal outcomes and that this relationship would be mediated by mother’s appraisal of the delivery and would attenuate over time.	Longitudinal cohort	United States	570	4 and 12 months postpartum	Unspecified	The Eysenck Personality Inventory Form, The Centre for Epidemiologic Studies Depression Scale, Rosenberg’s (1965) self-esteem scale	Mother-infant interactions, Neuroticism, Depression, Self-esteem, appraisal of the birth experience.	The psychological experiences associated with delivery by unplanned CS, by planned CS, or VD are distinct, and unplanned CS deliveries are appraised most negatively.	****
Eckerdal, 2017	To explore the association between MoD and postpartum depression.	Longitudinal cohort	Sweeden	3888	118th gestational week, the 32nd week of pregnancy, at 6 weeks, 6months postpartum	2009–2014	EPDS	Postpartum depression	A higher prevalence of depressive symptoms at 6 weeks postpartum was noted among women who delivered by EmCS, whereas no significant association with MoD was found regarding PPD at six months postpartum.	*****
Enabudoso, 2011	To assess the prevalence of satisfaction, and associated factors, among women who had recently delivered by CS.	Cross-sectional	Nigeria	211	2–5 days postpartum	2010	Study sepcific	Satisfaction	Satisfaction with CS was significantly higher among women who had ElCS as compared with EmCS.	***
Fenaroli, 2016	To explore the influence of cognitive and emotional variables on labour and delivery outcomes and examine how individual characteristics, couple adjustment, and medical factors influence the childbirth experience.	Longitudinal cohort	Italy	121	Between 32 and 37 weeks of pregnancy and 30–40 days postpartum	2010–2012	Wijma Delivery Expectancy Questionnaire, EPDS, Dyadic Adjustment Scale	Childbirth expectations, depression	There was no relationship found between MoD and perceived emotional experience.	****
Fenwick, 2009	To explore women’s experiences of CS.	Qualitative	England	21	Between 7 and 32 weeks postpartum	1999–2000		Experiences	Feelings of failure were present whether or not the CS was planned or an emergency, and these feelings had an impact on their status passage to motherhood for several reasons. The surgery resulted in the loss of women’s familiar, normal, healthy body. From their perspective, their body had let them down, denying them a normal birth.	*****
Forti-Buratti, 2017	To compare the mother-to-infant bond of mothers who gave birth by elective C-section versus EmCS.	Prospective cohort	Spain	116	48–72 h and 10–12 weeks after delivery	Not specified	Mother-to-Infant Bonding Scale, responses to separation	Mother-infant bonding	No significant differences between the two CS in bonding, newborn response to separation or type of feeding were observed at any time points.	****
Furuta, 2016	To identify factors associated with birth-related post-traumatic stress symptoms during the early postnatal period.	Prospective cohort	England	1824	6–8 weeks postpartum	2010	Impact of Event Scale	PTSD	EmCS was a high risk factor for post-traumatic stress symptoms.	*****
Gamble, 2005	To examine the relationship between MoD and symptoms of psychological trauma at 4–6 weeks postpartum	Prospective cohort	Australia	400	72 h and 4–6 weeks postpartum	2001–2002	Mini-International Neuropsychiatric Interview-Post-Traumatic Stress Disorder(MINI-PTSD)	PTSD	Women who had an EmCS or operative VD were more likely to meet the diagnostic criteria for PTSD than women who had an ElCS section or spontaneous VD.	****
Gaillard, 2014	To identify socio-demographic, psychosocial and obstetrical risk factors of postpartum depression.	Prospective cohort	France	312	32–41 weeks gestation, and6–8 weeks postpartum	2007–2009	EPDS (French version)	Depression	Women with PND did not differ from the others in MoD (spontaneous vaginal, assisted vaginal, EmCS or ECS).	****
Gibbins, 2001	To explore, describe and understand the expectations during pregnancy and subsequent experiences of childbirth in women.	Qualitative	England	8	2 weeks post birth	Unspecified	Study specific	Experiences	Women expressed positive feelings about their labours, even though all women felt that labour was different to what they had expected.	*****
Goker, 2012	To determine the effect of MoD on the risk of postpartum depression.	Cross-sectional	Turkey	318	6 weeks postpartum	Unspecified	EPDS	Depression	Delivering by spontaneous VD, ECS, or EmCS had no effect on EPDS scores.	***
Graham, 1999	To assess the degree and nature of women’s involvement in the decision to deliver by CS section, and women’s satisfaction with this involvement.	Qualitative	Scotland	166	3–4 days and 6–12 weeks postpartum	1995–1996	Study specific	Satisfaction and decision making	Women undergoing ElCS section generally received adequate information; however, with EmCS, half of the women had not received enough information during pregnancy. A significant proportion of women experienced negative feelings, particularly with EmCS (30%).	****
Guittier, 2014	To determine important elements associated with first delivery experience according to the MoD.	Qualitative	Switzerland	24	4–6 weeks postpartum	2012	Study specific	Experiences	The MoD directly impacted on key delivery experience determinants as perceived control, emotions, and the first moments with the newborn.	****
Handelzalts, 2017	To compare the impacts on childbirth experience of `planned’ delivery (elective CS and vaginal delivery) versus `unplanned’ delivery (vacuum extraction or EmCS).	Cross-sectional	Israel	469	Up to 72 h postpartum	2014–2015	Subjective Childbirth Experience Questionnaire and Personal Information Questionnaire	Experience	Unexpected MoD (EmCS) results in a more negative birth experience than a planned MoD.	*****
Herishanu-Gilutz, 2009	To examine the significance of the subjective experience of mothers who gave birth by an EmCS.	Qualitative	Finland	10	4–6 months	Unspecified	Study specific	Experiences	Themes were identified related to the traumatic experience of the operation, e.g. sense of loss of control regarding the decision to operate, feeling of fear and anger toward the caretaking staff.	*****
Hobbs, 2016	To examine MoD and breastfeeding initiation, duration, and difficulties reported by mothers at 4 months postpartum.	Prospective Cohort	Canada	3021	34–36 weeks gestation and 12–14 months postpartum	2008	Unspecified	Infant feeding	Women who delivered by EmCS had a higher proportion of breastfeeding difficulties (41%), and used more resources before (67%) and after (58%) leaving the hospital, when compared to VD (29, 40, and 52%, respectively) or planned CS (33, 49, and 41%, respectively).	****
Iwata, 2015	To identify factors for predicting post-partum depressive symptoms after childbirth in Japanese women.	Prospective Cohort	Japan	479	1 day before hospital discharge, 1, 2, 4, and 6 months post-partum.	2012–2013	EPDS, The Postnatal Accumulated Fatigue Scale, The Postpartum Maternal Confidence Scale, The Childcare Value Scale	Depression	Six variables reliably predicted the risk of postpartum depression including EmCS.	*****
Jansen, 2007	To investigate fatigue and HRQoL in women after VD, ElCS, and EmCs.	Prospective cohort	Netherlands	141	12–24 h after VD and 24-48 h after CS and 1,3, weeks postpartum	2003–2004	The Multidimensional Fatigue Inventory, EuroQoL 5D, Short-Form 36	HRQoL	Patients after VD had higher mean physical HRQoL scores than after CS. The average period to reach full physical recovery was 3 weeks after VD, 6 weeks after elective CS, and 6 weeks after EmCS.	*****
Karlström, 2017	To compare self-reported birth outcomes for women undergoing birth through spontaneous onset of labour between those who actually had a vaginal birth and those who eventually had an EmCS.	Prospective Longitudinal	Sweden	870	Mid pregnancy (18–19 weeks), late pregnancy (32–34 weeks), 2 months and 1 year postpartum/	Unspecified	Study specific	Birth fear and experience	Birth experience were more among women having an EmCS.	****
Karlstrom, 2007	To investigate women’s experience of postoperative pain and pain relief after CS and factors associated with pain assessment and the birth experience.	Cross-sectional	Sweden	60	2–9 days postpartum	2004 and 2005	The Visual Analog Scale, and study specific	Experiences	The risk of a negative birth experience was 80% higher for women undergoing an EmCS compared with elective CS.	***
Loto, 2010	To examine the association between the MoD, self-esteem, and parenting self-efficacy both at delivery and at 6 weeks postpartum.	Prospective cohort	Nigeria	115	Prior to hospital discharge and 6 weeks postpartum	2007–2008	Rosenberg self-esteem scale and parent–child relationship questionnaire	Self-esteem	Factors that were significantly associated with low self-esteem include being single and having EmCS.	***
Loto, 2009	To assess the level of self-esteem of newly delivered mothers who had CS andevaluate the sociodemographic and obstetrics correlates of low self-esteem in them.	Cross-sectional	Nigeria	109		2007–2008	Rosenberg self-esteem scale	Self-esteem	EmCS closely correlated with low self-esteem in women who had CS.	****
Lurie, 2013	To evaluate sexual behaviour longitudinally in the postpartum period by MoD.	Prospective cohort	Israel	82	6, 12, and 24 weeks postpartum	2010–2011	Female Sexual Function Index	Sexual Function	Sexual function did not differ significantly by MoD at 6, 12, or 24 weeks postpartum.	****
Maclean, 2000	To examine women’s distress in response to one of four obstetric procedures: spontaneous VD; induced VD; instrumental VD; or, EmCS.	Cross-sectional	England	40	6 weeks postpartum	1996–1997	Impact of Event Scale, Hospital Anxiety and Depression Scale	Experience, wellbeing, distress	Women who gave birth assisted by instrumental delivery reported the childbirth event as distinctly more distressing than the women in the other three obstetric groups (VD; induced VD; EmCS).	****
Modarres, 2012	To estimate the prevalence of childbirth-related post-traumatic stress symptoms and its obstetric and perinatal risk factors.	Cross-sectional	Iran	400	6–8 weeks after birth	2009	Post-traumatic Symptom Scale-Interview	PTSD	EmCS was a significant contributing factor to PTSD after childbirth.	****
Noyman-Veksler, 2015	To investigate the protective role of sense of coherence (SOC) and perceived social support in the effect of EmCS/ELCS on postnatal psychological symptoms and impairment in mother–infant bonding.	Prospective Longitudinal	Israel	142	6 and 12 weeks postpartum	Unspecified	Post-partum bonding questionnaire, Post-traumatic diagnostic scale, Edinburgh post-natal depression questionnaire, Sense of coherence, Social support questionnaire	Depression, bonding, PTSD, social support	No effect was found of the MoD on bonding with the infant. An EmCS predicted an increase in PTSD symptoms in Time 2, but only among women with low levels of Time-1 social support.	****
O’Reilly, 2014	To establish a greater understanding of the emotional and cognitive mechanisms associated with CS.	Cross-sectional	France	201	At least 6–8 weeks postpartum	2011–2012	Labour Agentry Scale, Maternal Self Report Inventory, Unconditional Self-AcceptanceQuestionnaire	Sense of control during the delivery, maternal self-esteem self-acceptance	Sense of control during labour and delivery was significantly higher for women who had a spontaneous VD when compared to those who had undergone an instrumental VD, a planned, or an EmCS.	*****
Patel, 2005	To assess the association between elective CS section and PD compared with planned VD and whether EmCS or assisted VD is associated with PD compared with spontaneous vaginal delivery.	Prospective cohort	UK	10,934	8 weeks postpartum	1991–1992	EPDS	Depression	No increased risk of PD was found between MoD.	*****
Porter, 2007	To explore the factors that women identified as distressing so as to understand their responses to standard questions on satisfaction.	Mixed methods	Scotland	1661	Up to 22 years postpartum	2002	Study specific	Distress	Many women had never had an operation before and the fact that their CS was classified as an “emergency” frightened them.	****
Redshaw, 2010	To gain a better understanding of CS by investigating women’s recent experiences and reflections on their care.	Qualitative	England	2960	3 months postpartum	2006	Study specific	Experiences with care	Fear and confrontation with the unexpected were themes identified from women who had an EmCS.	*****
Rowlands, 2012	To examine the physical and psychological outcomes of women in the first three months after birth, and whether these varied by MoD.	Cross-sectional	England	5332	3 months postpartum	2010	Study specific	PTSD and general psychological outcomes	Women having unplanned CS section births were marginally more likely to report PTSD-type symptoms, however, there was no association between PTSD type symptoms and planned CS section births.	****
Ryding, 1998	To describe women’s thoughts and feelings during the process of a delivery that ended in an EmCS, to ascertain if an EmCS might fulfil the stressor criterion PTSD according to DMS IV.	Qualitative	Sweden	53	2 days after birth	Unspecified	Study specific	PTSD and Experiences	55% of women experienced intense fear for their own life or that of their baby. 8% felt very badly treated by the staff. Almost all women had adequate knowledge of the reasons for the EmCS.	*****
Ryding, Wijma 1998	To compare the psychological reactions of women after EmCS, ElC, instrumental VD, and normal VD.	Prospective cohort	Sweden	326	2 days and 1 month postpartum	1992–1993	Wijma Delivery Expectancy Experience Questionnaire the Impact of Event Self-Rating ScaleI, 35-item version of the Symptoms Check List	Experiences and trauma	The EmCS group reported the most negative delivery experience at both times, followed by the lVD group. At a few days postpartum the EmCS group experienced more general mental distress than the VD group, but not when compared with the ElCS or the instrumental VD groups. At 1 month postpartum the EmCS group showed more symptoms of post-traumatic stress than the ECS and instrumental VD groups, but not when compared to the VD group.	****
Ryding, 2000	To investigate the possibility to categorize women’s experiences of EmCS based on the patterns displayed in their narration of the event, and to describe typical features of those categories.	Qualitative	Sweden	25	A few days and 1–2 months postpartum.	Unspecified	Study specific	Experiences	The narratives of the 25 women were categorized as follows: Pattern 1 - confidence whatever happens (n 5); Pattern 2 - positive expectations turning into disappointment (n 7); Pattern 3 - fears that come true (n 9); and Pattern 4 - confusion and amnesia (n 4).	*
Safarinejad, 2009	To quantify the relationship between MoD and subsequent incidence of sexual dysfunction and impairment of quality of life (QOL) both in women and their husbands.	Prospective cohort	Iran	912	Every month post deliveryup to 12 months.	2006–2007	Female Sexual Function Index (FSFI), and International Index of Erectile Function (IIEF),	Sexual Function, QoL	Women with VD and EmCS had statistically significant lower Female Sexual Function Index (FSFI) scores as compared with planned CS Section women	*****
Saisto, 2001	To examine the extent to which personality characteristics, depression, fear andanxiety about pregnancy and delivery, and socio-economic background, predict disappointment with delivery and the risk of puerperal depression.	Prospective Longitudinal	Finland	211	Once after the 30thweek of pregnancy, and 2–3 months after delivery	Unspecified	Beck’s Depression Inventory, the NEO-PI Scale for neuroticism, a partnership satisfaction scale, a Pregnancy Anxiety Scale, a revised version of a fear-of-childbirth questionnaire	Disappointment with delivery and satisfaction	Strongest predictors of disappointment with delivery were labour pain and EmCS.	*****
Sarah, 2017	To investigate the relationship between type of delivery and postpartum depression.	Cross-sectional	Iran	Unspecifed	Unspecified	2013	Beck depression inventory	Depression	The prevalence of postpartum depression is 33.4%, respectively, of which 13.8% related to EmCS, 7.2% of vaginal deliveries, and 8% of elective CS.	**
Shorten, 2014	To explore women’s values and expectations during their process of decision making about the next birth.	Qualitative	Australia	187	36–38 weeks pregnant and 6–8 weeks postpartum	Unspecified	Study specific	Decisions after prior CS	Women described long labours ending in CS did not want to go through it again, and especially did not want to repeat the “emergency” scenario. Many described a sense of loss after the previous CS experience and expressed a personal need to remedy this feeling through a better experience in the next birth. “After an emergency CS I felt I had failed, I felt cheated of the childbirth experience I had wanted”.	*****
Soderquist, 2002	To study whether or not a more stressful delivery was positively related to traumatic stress after childbirth.	Cross-sectional	Sweden	1550	Unspecified	1994–1995	Traumatic event scale	Traumatic stress	Traumatic stress symptoms and having a PTSD symptom profile were both significantly related to the experience of an EmCS or an instrumental VD.	****
Somera, 2010	To explore women’s experience of an EmCS birth to gain a better understanding of their thoughts, and feelings throughout the birth process.	Qualitative	Canadian	9	1–5 days after birth and 11–27 days after birth	Not specified	Open-ended questions	Experience	Seven themes were identified describing the women’s experience: (1) It was for the best, (2) I did not have control, (3) Everything was going to be okay, (4) I was so disappointed, (5) I was so scared, (6) I could not believe it and (7) I was excited.	*****
Spaich, 2013	To investigate the extent to which satisfaction with childbirth depends on the MoD, and evaluated factors determining postpartum satisfaction.	Prospective cohort	Germany	335	Unspecified	2010–2011	Salmon’s Item List	Experience	There were no women in the subgroup with EmCS who score indicating an overall negative birth experience. The subjective experience of birth was described as ‘good/very good’ in 89% of the women who underwent EmCS.	****
Storksen, 2013	To assess the relation between fear of childbirth and previous birth experiences.	Prospective cohort	Norway	1657	Weeks 17 and 32 pregnant	2009–2011	Wijma Delivery Expectancy Questionnaire	Fear	EmCS and vacuum extraction were associated with fear of childbirth in subsequent pregnancies.	*****
Tham, 2007	To examine the associations between new mother’s sense of coherence (SOC) and obstetric and demographic variables a few days postpartum, and post-traumatic stress symptoms 3 months’ postpartum in relation to women who had undergone an emergency CS section.	Prospective cohort	Sweden	122	2 days and 3 month postpartum	Not specified	Sense of Coherence Scale (SOC-13), Impact of Event Scale (IES-15).	PTSD	25% of the women reported symptoms of post-traumatic stress to a moderate degree (indicating a need for follow-up), and 9% had a high degree of symptoms (indicating possible PTSD).	****
Tham, 2010	To describe women with and without symptoms of post-traumatic stress following EmCS, and how they perceived the support received in connection with the birth of their child.	Qualitative	Sweden	84	6–7 months postpartum	Not specified	Questions seeking the women’sexperienced social and emotional support from the staffand from their families	Experience and support	The midwives’ action, the content and organisation of care, the women’s emotions, and the role of the family were main categories that seemed to influence the interviewees’ perceptions of support in connection with childbirth. Women with PTSS further mentioned nervous or non-interested midwives, intense fear and feelings of shame during delivery, lack of postnatal follow-up, long-term postpartumfatigue and inadequate help from husbands as influencing factors. Women without symptoms reported involvement in the EmCS decision and a feeling of relief.	****
Trivino-Juarez, 2017	To conduct a longitudinal study to analyse differences in HRQoL at the sixth week and sixth month postpartum, with mode of birth as the main independent variable.	Prospective Longitudinal	Spain	547	6 weeks and 6 months postpartum	2013–2014	EPDS, SF-36	HRQoL	Women who had vaginal, forceps or vacuum-extraction births at the sixth week postpartum reported better physical functioning than women who had elective or EmCS. At the sixth month postpartum, a significantly higher proportion of women in the forceps group (34%) than in the EmCS group (15%) reported being less satisfied with their sexual relations than before pregnancy.	****
Tully, 2013	To examine women’s experiences of and explanations for undergoing cesarean delivery.	Qualitative	England	115	Not specified	2006–2009	Study specific	Experiences	All mothers described labour prior to their unscheduled caesareans as wasted effort.	*****
Ukpong, 2006	To investigate postpartum emotional distress including depression women who had a CS by comparing them at 6–8 weeks following childbirth with 47 matched controls who had normal vaginal delivery.	Cross-sectional	Nigeria	94	6–8 weeks postpartum	Unspecified	General Health Questionnaire (GHQ-30), Beck Depression inventory	Depression, general health	There was no relationship between the depression scores and being scheduled for either ElCS or EmCS.	****
Vossbeck-Elsebusch, 2014	To replicate earlier findings regarding the prediction of PTSD levels following childbirth by known prenatal, perinatal and postnatal predictors.	Prospective cohort	Germany	224	1–6 months	Unspecified	Posttraumatic DiagnosticScale (PDS), University of California, Los Angeles Social SupportInventory (UCLA-SSI-d), Peritraumatic DissociativeExperience Questionnaire (PDEQ), PosttraumaticCognitions Inventory (PTCI), Responsesto Intrusions Questionnaire (RIQ), German version of the PerseverativeThinking Questionnaire (PTQ)	PTSD	The mean PDS (Posttraumatic Diagnostic Scale) score for women who had an EmCS were significantly higher than the PDS score for women who had a normal VD.	*****
Wijma, 2002	To examine whether the women’s psychological condition during pregnancy correlates with their psychological well-being after EmCS.	Prospective cohort	Sweden	1981	Gestation week 32, a few days, and one month	Unspecified	Wijma Delivery Expectancy/ Experience Questionnaire, Spielberger Trait Anxiety Inventory, Stress Coping Inventory, Impact of Event Scale, Symptom Checklist	Fear	Surgical complications including EmCs correlated with postpartum fear of childbirth negatively a few days after the operation, but positively one month later.	****
Wiklund, 2009	To examine changes in personality from late pregnancy to early motherhood in primiparas having vaginal or CS.	Prospective cohort	Sweden	314	37–39 gestational weeks in pregnancy and 9 months after delivery.	2003–2006	Karolinska Personality Scales	Personality	Women who had an EmCS scored higher on the subscale measuring Psychasthenia (low degree of mental energy and stress susceptible) 9 months after birth compared to those who had a spontaneous VD.	****
Wiklund, 2007	To examine the expectations and experiences in women undergoing a CS on maternal request and compare these with women undergoing CS with breech presentation as the indication and women who intended to have VD acting as a control group and to study whether assisted delivery and EmCS in the control group affected the birth experience.	Prospective cohort	Sweden	496	Prior to delivery and 3 months postpartum	2003–2005	Wijma Delivery Expectancy/Experience Questionnaire	Experiences	Women planning a VD but experiencing an EmCS or an assisted VD had more negative birth experiences than the other groups.	****
Xie, 2011	To examine whether or not CS delivery is associated with increased risk of postpartum depression.	Cross-sectional	China	534	2 weeks postpartum	2007	Chinese version of the EPDS (EPDS), Social Support Rating Scale,	Depression	PPD rate was higher in the group who had elective CS delivery than inthe group who had EmCS.	****
Yang, 2011	To examine whether MoD are associated with postnatal depression.	Prospective cohort	Taiwan	10,535	Unspecified	2003–2006	Data collected from the National Health Insurance Research Database	Depression	Risk of acquiring PPD was lower in mothers with a normal VD or an instrumental VD compared to mothers with an EmCS. The women who elected to have a CS section was higher risk than an EmCS.	****
Zanardo, 2016	To assess feelings towards newborn infants in mother swho delivered by elective (ElCD) or emergency EmCS.	Cross-sectional	Italy	573	Not specified	2014–2015	Mother-to-Infant Bonding Scale (MIBS)	Mother-infant bonding	EmCS negatively affected mother bonding and opening emotions, and originated inmother feeling sadness and disappointment for the unplanned delivery.	**

^a^Mixed Methods Appraisal Tool Quality Assessment Rating

### Quality assessment

Mixed Methods Appraisal Tool quality assessment ratings (MMAT QARs) are included in Table 1. Among the 51 quantitative non-randomised studies, 14 met all five criteria, 31 met four criteria, 4 met three criteria and 2 met two criteria. Of the 14 qualitative studies, 12 met all five criteria. The one study with mixed methods met four of the five criteria. The main reason several quantitative studies did not meet all criteria was a lack of reporting for the complete set of outcomes (without adequate justification), response rate or follow-up rate.

### Data extraction and synthesis

Key psychosocial outcomes were examined in the final 66 studies. Data synthesis was employed to extract and synthesise data pertaining to key psychosocial outcomes from each study into coherent themes. Psychosocial outcomes potentially associated with EmCS included postpartum depression, post-traumatic stress, health related quality of life, mother infant bonding, infant feeding, sexual function, experiences, satisfaction, self-esteem, distress, and fear. Due to an excess of methodological heterogeneity between studies (even for subsets of studies with some common features), a meta-analysis was deemed inappropriate. Table 2 summarizes evidence of associations for identified psychosocial outcomes and EmCS.

**Table 2:** Associations of identified psychosocial outcomes and EmCS

Key psychosocial outcomes	Number of studies	Association between EmCS and psychosocial outcomes	Inconclusive associations between EmCS and psychosocial outcomes	Qualitative summary
Postpartum depression (PPD)	12		+	Studies reported inconsistent findings. The majority of studies reported no significant association (*n* = 7) between EmCS and PPD whereas the remaining studies reported a relationship between EmCS and increased symptoms of PPD (*n* = 5).
Post-traumatic stress disorder (PTSD)	11	+		All studies (*n* = 11) reported consistent findings that EmCS was a contributing factor to increasing post-traumatic stress symptoms and PTSD after childbirth.
Health related quality of life	2	–		Consistent findings were found across studies (*n* = 2) that women who had an EmCS had poorer physical functioning compared to other MoDs.
Mother infant bonding	3		–	Studies reported inconsistent findings. In *n = 1* study EmCS appeared to have a negative association with mothers bonding and opening emotions with their baby. In contrast, no significant affect was found in terms of MoD on mother-infant bonding in the remaining studies (*n* = 2).
Infant feeding	3	–		Consistent findings were found across studies in that EmCS impacted negatively in varying ways on infant feeding (*n* = 3). Women who have an EmCS were more likely to have had an unsuccessful first breastfeeding attempt, were less likely to breastfed their baby within the first 24 h and upon leaving the hospital, and to breastfeed for a shorter duration of time compared to other MoDs.
Sexual function	3		+/−	Studies were inconsistent in their findings (*n* = 3) in terms of satisfaction with sexual relations after birth and sexual function postpartum.
Experiences	21	+/−		In terms of quantitative research (*n* = 9), the majority of studies found that EmCS was more likely to result in a negative birth experience (*n* = 6), *n* = 1 study reported MoD had no influence on mother experiences and *n* = 2 studies reported that EmCS was related to positive experiences in comparison to other MoDs. In terms of the qualitative studies (*n* = 12) women described a wide variety of emotions as salient aspects to their EmCS experience however, a number of dominating negative experiences were consistent across all studies
Satisfaction	4	–		Consistent findings were reported across all studies (*n* = 4) with women who had an EmCS more likely to appraise their deliveries less favourably than those who delivered via other MoDs.
Self-esteem	3	–		Consistent findings were reported across all studies (*n* = 3). Women who had an EmCS were more likely to report feelings of emotional vulnerability after delivery including feelings of failure, regret, and lower self-esteem.
Distress	3		–	Findings were inconsistent in terms of distress after EmCS. No significant association between MoD and distress were reported in a study (*n* = 1), another study reported other MoD causing more distress than EmCS (*n* = 1), the final study reported a relationship between EmCS and distress.
Fear	2		–	Inconsistent findings were reported. With *n* = 1 study reporting EmCS was associated with increased fear of childbirth in subsequent pregnancies and *n* = 1 study reporting a correlation with fear of childbirth a few days after the operation, however this decreased one month later.
Other				
Childbirth Burden	1	+		Women who experienced emergency surgical intervention (i.e EmCS) were more likely to demonstrate higher childbirth burden scores than any other MoD (*n* = 1).
Feelings of control	1	–		Women who had a spontaneous VD reflected having a significantly higher sense of control during their labour and childbirth relative to with an instrumental VD, a planned CS, or an EmCS (*n* = 1).

+ indicates that some (or all) evidence supports a positive association

- indicates that some (or all) evidence supports a negative association

### Key outcomes

#### Postpartum depression

Twelve studies examined depression as an outcome of EmCS [33, 36, 38, 43, 45, 51, 60, 62, 71, 80, 85, 87]. These studies used varying measures, with the majority (*n* = 8) utilising the Edinburgh Postnatal Depression Scale (EPDS), three using Beck’s Depression Inventory (BDI) and one study not specifying the measure used. Studies identified reported mixed findings in terms of postpartum depression (PPD) and the experience of EmCS. The majority of studies found no significant association between having an EmCS and PPD relative to other MoDs [33, 38, 43, 45, 62, 80, 85]. For example, a prospective cohort study (*n =* 10, 934) from the UK found no significant evidence of increased risk of PPD between different MoDs including EmCS [62]. In contrast, a much smaller prospective cohort study reported EmCS was a predictor of PPD [51]. Additionally, a recent cross-sectional study conducted in Iran [71] reported that the prevalence of PPD was 33.4%, of which the highest proportion consisted of women who had experienced EmCS at 41.3%. Furthermore, a recent large longitudinal study found that compared with spontaneous VD, women who delivered by EmCS had significantly higher odds of PPD 6 weeks after delivery (OR = 1.45) [36]. Additionally, a cohort study (*n =* 10, 535) reported that the odds of PPD was significantly lower for women who had a normal VD (OR = 0.67) or an instrumental VD (OR = 0.56) compared to women who had EmCS [87]. However, women who had an elective CS had higher odds of PPD than women who had EmCS (OR = 1.48, *p* = 0.0168) [87]. Heterogeneity in the tools, their use and findings can be seen in Table 3 and makes the comparison of these figures problematic.

**Table 3:** Heterogeneity across studies examining depression

Study	Cut score	Time post partum	Sample size	Participants with depression	EmCS subgroup	EmCS subgroup with depression	Evidence of association between EmCS and PPD
Edinburgh Postnatal Depression Scale							
Eckerdal, 2017	EDPS> 12	6 weeks	3888	505 (13%)	346	50 (16.7%)	No
Gaillard, 2014	EDPS> 12	6–8 weeks	264	44 (16.7%)	44	6 (13.6%)	No
Goker, 2012	EDPS> 13	6 weeks	318	100 (31.4%)	106	37 (34.9%)	No
Iwata, 2015	EDPS> 9	6 months	479	21.5%	60	24 (40%)	Yes
Patel, 2005	EDPS> 13	8 weeks	10,934	N/A	572	56 (9.8%)	No
Xie, 2011	EDPS> 13	2 weeks	534	103 (19.3%)	149	24 (16.1%)	Yes: PPD higher in ElCS than EmCS
Beck Depression Inventory							
Chen, 2002	BDI 9–10	6 weeks	357	N/A	N/A	N/A	No
Sarah, 2017	N/A	N/A	N/A	33.4%,	N/A	13.8% of 33.4%	No mention
Ukpong, 2006	BDI > 9 significant, 10–18 mild/moderate, 19–29 moderate/severe, 30–63 extreme	6–8 weeks	47	29.8%	40	N/A	No

#### Traumatic stress

Eleven included studies examined trauma as an outcome of an EmCS [24, 34, 41, 42, 59, 60, 65, 66, 73, 76, 81]. These studies were conducted across a diverse range of countries including Australia, Nigeria, UK, Iran, Israel, Sweden and Germany. Study designs included, six cross-sectional, four prospective and one qualitative. All studies consistently reported that EmCS was a contributing factor for post-traumatic stress symptoms and Post Traumatic Stress Disorder (PTSD) after childbirth. Several of the studies stated that any unplanned interventions during childbirth including EmCS were predictors of PTSD [42, 88]. For example, a prospective cohort study (*n =* 1824) identified EmCS as a risk factor for post-traumatic stress symptoms [41]. Findings from a smaller cross-sectional study in Australia reported a greater than expected frequency of PTSD in women who had EmCS, specifically, 73% reporting trauma symptoms 4–6 weeks postpartum [42]. Further, a qualitative research study conducted in Sweden concluded that experiences of women who delivered via EmCS were traumatic enough to fulfil the stressor criterion of PTSD in the DSM IV [66]. This study stated that 55% of women interviewed a few days after an EmCS reported feelings of intense fear of death or injury to themselves or to their baby during the delivery process [66].

#### Health related quality of life

Two studies specifically examined Health Related Quality of Life (HRQoL) [52, 78]. One study utilised the Short-Form 36 (SF-36) to measure HRQoL [78] and the other utilised the SF-36 and the EuroQoL 5D [52]. Both studies reported consistent findings that women with an EmCS had poorer physical functioning, relative to other MoDs. A prospective study in the Netherlands reported that the average period to reach full physical recovery was 3 weeks after VD, 6 weeks after elective CS and EmCS [52]. Similarly, a larger more recent study reported that women who had a vaginal, forceps or vacuum-extraction delivery, had better physical functioning at 6 weeks postpartum relative to those with elective CS or EmCS [78]. In a cohort study in Sweden, women who had EmCS scored higher on the subscale measuring Psychasthenia (low degree of mental energy and stress susceptible) 9 months after birth relative to those with spontaneous VD [84].

#### Mother-infant bonding

Three studies examined the relationship between EmCS and mother-infant bonding [5, 35, 40] with conflicting results. Two studies utilised the Mother-to-Infant Bonding Scale [5, 40] and the third utilised the Parent-Child Early Relational Assessment Tool [35]. A recent, large scale cross-sectional study found EmCS appeared to have a negative association with mothers bonding and opening emotions with their baby. In contrast, a similar sized study reported no significant differences in mother-infant interactions at 4 or 12 months postpartum between MoD [35]. Similarly, a smaller scale cohort study found that type of CS did not appear to significantly affect mother-infant bonding in the first 72 h following delivery or at 12 weeks postpartum [40].

#### Infant feeding

Three studies examined the relationship between infant feeding and EmCS [25, 26, 50]. Study designs were prospective cohort, cross-sectional, and qualitative. The large scale prospective cohort study reported that women with EmCS were more likely to have an unsuccessful first breastfeeding attempt and were less likely to breastfed their baby within the first 24 h and upon leaving the hospital [50]. Furthermore, the study reported that women with EmCS had more breastfeeding difficulties (41%), and used more hospital resources before and after leaving the hospital (67, 58%), in comparison to those with a VD (29, 40, and 52%, respectively) or a planned CS (33, 49, and 41%, respectively). Additionally, a similar sized cross-sectional study reported that breastfeeding duration varied substantially with MoD [25]. In the same study, median breastfeeding duration was 45.2 weeks among women who had a spontaneous VD, 38.7 weeks among planned CS, 25.8 weeks among induced VD and 21.5 weeks among women with EmCS [25]. In the qualitative study women frequently stated that their decision to breastfeed was driven by their desire to make up for the traumatic way their baby was delivered, including, by EmCS [26]. In this study a women with EmCS stated, “breastfeeding became almost an act of vindication. I had to make up for failing to provide my daughter with a normal birth, so I sure wasn’t going to fail again” [26].

#### Sexual function

Three studies, conducted in Israel, Iran and Spain, examined the relationship between EmCS and sexual function postpartum [57, 69, 78], with inconsistent findings. A prospective cohort study reported a significantly higher proportion of women at 6 months postpartum being less satisfied with their sexual relations after birth in the forceps group (34%) relative to the EmCS group (15%) [78]. In contrast, a larger prospective cohort study reported that women who had a VD or EmCS had statistically significantly lower Female Sexual Function Index (FSFI) scores on average relative to those with a planned CS [69]. These findings were contrary to that of a small scale cohort study that found no significant difference between average sexual function scores and various MoD postpartum [57], potentially due to a lack of power.

### Experiences

A large number (*n* = 21) of identified studies examined women’s experiences with EmCS. A variety of measures were used across studies including: Impact of Event Scale, Wijma Delivery Expectancy/Experience Questionnaire, and Questionnaire for Assessing Childbirth Experience (QACE). Studies examined varying aspects of women’s experiences of EmCS including women’s overall birth experiences, emotional experiences and experiences with care and staff.

The majority of quantitative research studies found that EmCS was more likely to result in a negative birth experience. For example, a recent large prospective cohort study in Sweden reported that birth experience was more likely to be negative among women with EmCS relative to VD [53]. Similar findings were reported in another recent but smaller cross-sectional study, where unexpected MoD including EmCS resulted in a higher likelihood of negative birth experiences [48] with this finding supported in numerous other studies [32, 54, 83, 89]. Contrary to this finding, two prospective cohort studies reported that MoD had no direct influence on women’s experience of childbirth [38, 74]. Interestingly, in one of these studies no women in the EmCS subgroup attained a score which indicated a negative birth experience; rather 89% of these women described the birth experience as ‘good/very good’ [74]. Furthermore, the majority of women in this study with EmCS also evaluated their feelings of control during labour and the opportunities they had to make informed choices/decisions as ‘good/very good’ [74]. Interestingly, a large prospective study found that women who had a planned CS scored significantly lower in terms of negative birth perception than those who had an EmCS or a VD [30].

Twelve studies utilised a qualitative design to examine women’s experiences of an EmCS [9, 31, 39, 44, 47, 49, 64, 66, 68, 72, 77, 79]. In all of these studies, women described a wide variety of emotions as salient to their EmCS experience however, a number of dominating negative experiences were consistent across all studies including: loss of perceived control and feelings of helplessness [9, 31, 39, 47, 49]; fear (own or/and for baby) [9, 31, 64, 66, 68, 77]; and disappointment [9, 66, 77]. In a study conducted by Shorten [72] one participant reported “after an emergency caesarean I felt I had failed, I felt cheated of the childbirth experience I had wanted”.

#### Experiences with maternity care and staff

A large prospective cohort study reported that women who had an unplanned CS were more likely to indicate that they had received “less than good” midwifery care during childbirth [90]. It was suggested that as women who have an EmCS often have their care transferred to other care providers during childbirth, it is possible that the discontinuity of care between the providers may influence women’s experiences with staff [90].

#### Satisfaction

Four studies examined women’s satisfaction after EmCS [28, 37, 46, 70] with all reporting that women with EmCS were more likely to appraise their deliveries less favourably than those with other MoDs. In a large prospective cohort study conducted in both the Netherlands and England, EmCS appeared to be a contributing factor to a negative appraisal of birth [28].

#### Self esteem

Three studies examined women’s self-esteem and EmCS [32, 55, 56] with all studies reporting consistent findings. A cross sectional study reported that MoD influenced women’s mood at one-month postpartum, with an item reading ‘I am proud of myself’, representing self-esteem, being more likely to have negative results for women with EmCS [32]. In two smaller Nigerian studies, women were more likely to report feelings of emotional vulnerability after delivery including feelings of failure, regret, and lower self-esteem [55, 56].

#### Distress

Three studies in Norway, Scotland and England examined distress in relation to EmCS [23, 58, 63]. In a very large prospective cohort study (*n =* 55,814) conducted over a 10 year period, no significant association between MoD and emotional distress postpartum was reported [23]. Further, a small cross-sectional study reported that women who gave birth assisted by instrumental delivery were more likely to report that their birth was distinctly more distressing than women in three other obstetric groups (VD, induced VD, EmCS) [58]. A mixed methods study reported that the fact that a CS was classified as an “emergency” frightened women, resulting in feelings of distress [63].

#### Fear

Two studies examined fear as an outcome of EmCS [75, 82]. A large prospective cohort study reported that EmCS was associated with increased fear of childbirth in subsequent pregnancies [75]. A similarly designed and sized study found that EmCS correlated with increased postpartum fear of childbirth a few days after the operation, however this decreased 1 month later [82].

#### Other outcomes

Childbirth burden and feelings of control were examined in two studies. A large cross-sectional study reported that women who experienced emergency surgical intervention (EmCS and vacuum extraction) were more likely to demonstrate higher childbirth burden scores than those with any other MoD [29]. A small cross-sectional study reported that women who had a spontaneous VD had a significantly higher sense of control during their labour and childbirth relative to those with an instrumental VD, a planned CS, or an EmCS [61].

## Discussion

### Summary of findings

A number of psychosocial outcomes were consistently and negatively reported to be associated by EmCS including post-traumatic stress, HRQoL, infant feeding, experiences, satisfaction and self-esteem. All studies examining post-traumatic stress consistently found that EmCS was a contributing factor for symptoms and PTSD after childbirth. Two studies exploring HRQoL reported consistent findings that women with EmCS had poorer physical functioning relative to other MoDs. Three studies examining infant-feeding reported that women with EmCS were more likely to have an unsuccessful first breastfeeding attempt, less likely to breastfed within the first 24 h and upon leaving the hospital, and to breastfeed for a shorter duration of time in comparison to other MoDs. These results are consistent with those reported by Ahluwalia [25] who noted that women with EmCS often experience; a difficult labour, stress, and delays in mother-infant interactions, each of which may reduce the likelihood or duration of breastfeeding.

Consistent findings were reported for satisfaction in that women with EmCS were more likely to appraise their deliveries less favourably than those with other MoDs. Studies examining self-esteem found women who had an EmCS were more likely to report feelings of emotional vulnerability after delivery including feelings of failure, regret, and lower self-esteem. Twenty one articles examined varying aspects of women’s experiences of EmCS, which constituted the most commonly examined psychosocial outcome among included studies. In both quantitative and qualitative studies it was reported that women with EmCS were often at the highest risk of assessing their childbirth experience in a negative way and described a wide variety of negative emotions including: loss of perceived control and feelings of helplessness, fear (own or/and for baby), and disappointment.

Psychosocial outcomes including depression, mother-infant bonding, sexual function, fear, and distress were also identified and examined within in the literature. However, studies either reported mixed findings or no sufficient evidence of an association between these outcomes and EmCS.

### Limitations

We recognise that potentially relevant articles could have been missed, written in languages other than English, or indexed in other databases other than those chosen and therefore may not have been identified. Studies identified in the review were conducted in 22 diverse countries and as such it must be acknowledged that cross-cultural differences are common and can greatly influence women’s psychosocial outcomes of childbirth [91]. Postnatal access to healthcare; procedural differences; quality of available care; levels of social support; religious beliefs; poverty; societal attitudes regarding pregnancy, birth and motherhood; gender roles and attitudes regarding mental health problems are just a few of the known socio-cultural and environmental factors that may influence findings in the identified studies [92].

Of the included articles the strengths and meaningfulness of the findings differ substantially due to variations in study design, sampling procedures, and sample size. It has been previously identified that research examining the psychosocial outcomes of CS have generally suffered from numerous methodological limitations including; reliance on small sample sizes, use of measures of unknown reliability and validity and the lack of a comparison group or varying comparison groups [93]. Several of these limitations were present in the included studies. For example, as noted previously, one of the primary reasons for excluding articles was the failure to specify or differentiate between type of CS for women in a study. Furthermore, there was often no discussion within included studies about reasons and causes for EmCS and it is possible that some causes are more strongly associated with the psychosocial outcomes examined. Studies identified in the review reported on wide varying time frames for postpartum data collection, with collection ranging from hours after birth to years after birth as well ultilising different cut-points on the same measures for diagnosis. The timing of data collection is an important methodological consideration as there is considerable evidence that the impact of a women’s birth experience changes over time [94]. As time passes, the positive affect from one’s baby and satisfaction with being a mother has been shown in some cases to favourably influence a women’s feeling about her labour experience [94].

As a result of the heterogeneous nature of these factors (exemplified in Table 3 for depression), meaningful pooled quantitative measures of study findings were unable to take place, even for subsets of studies. Overall, there appears a paucity of published evidence with consistent measures and adherence to guidelines for reporting (e.g. for cut-scores) which is crucial to rectify in future studies so that (gold standard) systematic literature reviews can meaningfully pool data in a quantitative manner.

### Strengths and implications

To our knowledge, this study is the first to systematically review the available literature on women’s psychosocial outcomes of EmCS. The review presents the findings of quantitative, qualitative and mixed methods studies from a vast array of countries and as a result identifies and examines a wide variety of psychosocial outcomes.

The review has highlighted the need for the further development of technologies and clinical practices to reduce the number of unnecessary EmCSs. Critically, it underscores the requirement for evidence based strategies to provide psychosocial support and information about EmCS in the context of routine antenatal and postnatal care. While high-level research currently exists in this area, for example in the form of routine debriefing to prevent psychological trauma after childbirth (103), it fails to show benefit. More broadly, while programs for postnatal psychosocial support have been promoted in many countries to improve maternal knowledge related to parenting, mental health, quality of life, and physical health, it has been concluded in a systematic review that the most effective strategies remain unclear [95].

## Conclusion

The review has highlighted the diverse impact that EmCS can have on women. Numerous psychosocial outcomes that are negatively impacted by this MoD were identified including post-traumatic stress, health-related quality of life, experiences, infant-feeding, satisfaction, and self-esteem. In particular, there was strong consensus that EmCS contributes to symptoms and diagnosis of post-traumatic stress. This review has also highlighted the need for further investigation on this topic using robust methodology including the use of consistent, valid and reliable measures with consistent use of guidelines for appropriate cut scores, consistent comparison groups, adequately powered studies and differentiation between types of CS. Overall, enhanced knowledge and understanding in this area will provide an imperative step towards implementing effective strategies to improve women’s health and well-being following EmCS.

## Supplementary information

**Additional file 1.** Logic Grids.

## Data Availability

Not applicable.

## Abbreviations

BDI: Beck’s Depression Inventory
CS: Caesarean Section
EmCS: Emergency Caesarean Section
EPDS: Edinburgh Postnatal Depression Scale
HRQoL: Health Related Quality of Life
MMAT: Mixed Methods Appraisal Tool
MoD: Mode of Delivery
PPD: Postnatal depression
PROSPERO: Prospective register of systematic reviews
PRSIMA: Preferred Reporting Items for Systematic Reviews and Meta-analyses
PTSD: Post Traumatic Stress Disorder
QAR: Quality Assessment Rating
SF-36: Short-Form 36
VD: Vaginal delivery
