# Supplementary material for: Women’s psychosocial outcomes following an emergency caesarean section: A systematic literature review
Source: BMC Pregnancy Childbirth. 2019 Dec 30;19:535. doi: 10.1186/s12884-019-2687-7 (PMC6937939; doi:10.1186/s12884-019-2687-7)
Supplement: Supplementary file 1 — Additional file 1. Logic Grids. [file 12884_2019_2687_MOESM1_ESM.docx]

**Logic Grids**

**Scopus:**

(( "Emergenc* caesarean*"  OR  "emergenc* caesarean*"  OR  "Emergenc* Birth" )  AND  ( psychology  OR  depression  OR  "Postnatal depression"  OR  "Postpartum depression"  OR  "Baby blues"  OR  anxiet*  OR  "Social adjustment"  OR  adjustment*  OR  "Adjustment disorder*"  OR  adaptation  OR  "social function*"  OR  "Quality of life"  OR  "Life quality"  OR  "Health-related quality of life"  OR  hrqol  OR  qol  OR  "Breast feed*"  OR  "Breast fed"  OR  "Mental health"  OR  "Mental disord*"  OR  "Social support"  OR  "Psychosocial Support Systems"  OR  "Maternal behav*"  OR  "Maternal-fetal relations*"  OR  "Mother-child relations*"  OR  "Adverse effect*"  OR  morbidit*  OR  "Stress Disorder*"  OR  traumatic  OR  "Psychological stress*"  OR  "Posttraumatic stress disorder*"  OR  ptsd  OR  trauma  OR  wellbeing  OR  "Mood disorder*"  OR  emotion*  OR  regret*  OR  feeling*  OR  affectiv*  OR  "Emotional adjustment"  OR  "Emotional adaptation*"  OR  "Patient satisfaction"  OR  "Personal satisfaction"  OR  "Patient preference"  OR  "Maternal health"  OR  "Maternal welfare"  OR  "Sexual health" ))

**Pubmed:**
(((Emergency caesarean[tw] OR emergency cesarean[tw] OR ((cesarean[tw] OR caesarean[tw] AND emergency[tiab] OR emergencies[tiab])) OR "cesarean section"[mh:noexp] OR Emergency Birth[tw]))) AND ("Psychology"[mh] OR Psychology[tw] OR "Depression, postpartum"[mh] OR Postnatal depression[tw] OR Postpartum depression[tw] OR Post partum depression[tw] OR Post natal depression[tw] OR Baby blues[tw] OR "Anxiety"[mh] OR Anxiety[tw] OR Anxiet*[tw] OR "Social adjustment"[mh] OR Social adjustment[tw] OR Adjustment*[tw] OR "Adjustment disorders"[mh] OR Adjustment disorder[tw] OR Adaptation[tw] OR social function*[tw] OR "Quality of life"[mh] OR Quality of life[tw] OR Life quality[tw] OR Health-related quality of life[tw] OR HRQOL[tw] OR QOL[tw] OR "Breast feeding"[mh] OR Breast feeding[tw] OR Breastfeeding[tw] OR "Mental health"[mh] OR Mental health[tw] OR "Mental disorders"[mh:noexp] OR Mental disord*[tw] OR "Social support"[mh] OR Social support*[tw] OR "Psychosocial Support Systems"[mh] OR Psychosocial Support Systems OR "Maternal behaviour"[mh] OR Maternal behave*[tw] OR "Maternal-fetal relations"[mh] OR Maternal-fetal relations[tw] OR "Mother-child relations"[mh] OR Mother-child relations[tw] OR Adverse effect*[tw] OR "Postpartum period"[mh] OR Postpartum period[tw] OR "Morbidity"[mh] OR Morbidity[tw] OR Stress Disorders, Traumatic[mh] OR "Stress, Psychological"[mh] OR Stress disorder[tw] OR Posttraumatic stress disorders[tw] OR Post traumatic stress disorder[tw] OR Trauma[tw] OR Wellbeing[tw] OR Well-being[tw] OR "Mood disorders"[mh] OR Mood disorders[tw] OR "Emotions"[mh] OR Emotions[tw] OR Regret*[tw] OR Feelings[tw] OR Affectiv*[tw] OR "Emotional adjustment"[mh] OR Emotional adjustment[tw] OR Emotional adaptation*[tw] OR "Patient satisfaction"[mh] OR Patient satisfaction[tw] OR "Personal satisfaction"[mh] OR personal satisfaction[tw] OR Patient preference[tw] OR "Maternal health"[mh] OR Maternal health[tw] OR "Maternal welfare"[mh] OR Maternal welfare[tw] OR "Sexual health"[mh] OR Sexual Health[tw])

**Embase:**

“cesarean section”/exp AND ((caesarean OR cesarean) NEAR/5 emergenc*):ti,ab

AND

“maternal disease”/de OR “maternal disease*”:ti,ab OR “perinatal depression”/de OR “perinatal depression”:ti,ab OR “postpartum depression”:ti,ab OR “postnatal depression”/de OR “postnatal depression”:ti,ab OR “baby blues”:ti,ab OR anxiety*:ti,ab OR “social adaptation”/de OR “social adaptation”:ti,ab OR “adjustment disorder”/de OR “quality of life”/de OR “quality of life”:ti,ab OR “health related quality of life”:ti,ab OR qol:ti,ab OR hrql:ti,ab OR “breast feeding”/de OR “breast feed*”:ti,ab OR “mental health”/de OR “mental health”:ti,ab OR “social support”/de OR “social support”:ti,ab OR “psychosocial support”:ti,ab OR “psychosocial care”/de OR “maternal behavior”/de OR “maternal behavior*”:ti,ab OR “maternal behaviour*”:ti,ab OR “maternal care”:ti,ab OR “mother fetus relationship”/de OR “mother fetus relation*”:ti,ab OR “mother foetus relation*”:ti,ab OR “mother child relation*”:ti,ab OR puerperium/de OR puerperium:ti,ab OR “maternal morbidity”/exp OR “maternal morbidit*”:ti,ab OR “posttraumatic stress disorder”/de OR “traumatic stress”:ti,ab OR “posttraumatic stress”:ti,ab OR “mental stress”/de OR “mental stress”:ti,ab OR “psychological well-being”/de OR “psychological well-being”:ti,ab OR wellbeing/de OR wellbeing:ti,ab OR “mood disorder”/de OR “mood disorder*”:ti,ab OR emotion/de OR emotion* OR regret* OR “psychological adjustment”/de OR “psychological adjustment”:ti,ab OR “patient satisfaction”/de OR “patient satisfaction”:ti,ab OR “maternal care”/de OR “maternal care”:ti,ab OR “maternal welfare”/de OR “maternal welfare”:ti,ab OR “sexual health”/de OR “sexual health”:ti,ab

**PsychInfo**

Emergenc* caesarean .ti,ab OR emergenc* cesarean .ti,ab OR Exp Caesarean Birth OR Emergency Birth .ti,ab

AND

Psychology.sh OR Psychology .ti,ab OR Exp Health Care Psychology OR Health Care Psychology.ti,ab
OR Exp Postpartum depression OR Postpartum depression .ti,ab OR Post partum depression .ti,ab OR Postnatal depression .ti,ab OR Post natal depression .ti,ab OR Baby blues .ti,ab OR Exp Anxiety OR Anxiet* .ti,ab OR Exp Social adjustment OR Social adjustment .ti,ab OR Adjustment* .ti,ab OR Exp Adjustment disorders OR Adjustment disorder .ti,ab OR Adaptation .ti,ab OR Social function* .ti,ab OR Exp Social Facilitation Exp Quality of life OR Quality of life .ti,ab OR Life quality .ti,ab OR Health-related quality of life .ti,ab OR HRQOL .ti,ab OR QOL .ti,ab OR Exp Breast feeding OR Breast feeding .ti,ab OR Breastfeeding .ti,ab OR Exp Mental health OR Mental health .ti,ab OR Exp Mental disorders OR Mental disord* .ti,ab OR Exp Social support OR Social support* .ti,ab OR OR Psychosocial Support Systems.ti,ab OR Exp Psychosocial Readjustment OR Psychosocial Readjustment.ti,ab OR Exp Mother Child Relations OR Mother Child Relations.ti,ab OR Maternal behav* .ti,ab OR Mother-child relations .ti,ab OR OR Exp Postpartum Psychosis Postpartum psychosis.ti,ab OR Postpartum period ti,ab OR Exp Morbidity OR Morbidity .ti,ab OR Exp Stress OR Stress.ti,ab OR ExpStress and Coping Measures OR Exp.Psychological stress OR Psychological stress.ti,ab OR Stress disorder .ti,ab OR Exp Posttraumatic Stress Disorder OR Posttraumatic stress disorders .ti,ab OR Post traumatic stress disorder .ti,ab OR Exp.Trauma OR Trauma .ti,ab OR Exp. Well Being OR Wellbeing .ti,ab OR Well-being .ti,ab OR Exp Affective Disorders OR Affective Disorder .ti,ab Mood disorders .ti,ab OR Exp Emotions OR Emotions .ti,ab OR Exp Regret OR Regret* .ti,ab OR Feeling* .ti,ab OR Affectiv* .ti,ab OR Exp Affective Disorders OR Affective Disorders.ti,ab OR Exp Emotional adjustment OR Emotional adjustment .ti,ab OR Emotional adaptation* .ti,ab OR Exp Satisfaction OR Satisfaction ti,ab OR Exp Client Satisfaction OR Client Satisfaction ti,ab OR Patient satisfaction ti,ab OR Personal satisfaction ti,ab Patient preference .ti,ab OR Maternal health .ti,ab OR Maternal welfare .ti,ab OR Sexual Health .ti,ab
